# Prevalence and impact of sarcopenia on urinary incontinence in localized prostate cancer patients undergoing laparoscopic radical prostatectomy

**DOI:** 10.3389/fruro.2025.1567575

**Published:** 2025-04-14

**Authors:** Tengfei Gu, Jie Li, Dan Wu, Ting Chen, Yongtao Pan, Qinzhou Yu, Jing Sha

**Affiliations:** ^1^ Department of Urology, Lishui Municipal Central Hospital, The Fifth Affiliated Hospital of Wenzhou Medical University, Lishui, China; ^2^ Department of Nursing, Lishui Municipal Central Hospital, The Fifth Affiliated Hospital of Wenzhou Medical University, Lishui, China

**Keywords:** prostate cancer, laparoscopic radical prostatectomy, sarcopenia, urinary incontinence, risk factor

## Abstract

**Objective:**

This research seeks to assess the prevalence of sarcopenia in patients diagnosed with localized prostate cancer and to investigate the influence of sarcopenia on the incidence of urinary incontinence following laparoscopic radical prostatectomy.

**Methods:**

A cohort of 350 patients, diagnosed with prostate cancer and having undergone laparoscopic radical prostatectomy at our hospital’s urology department between January 2022 and December 2023, was selected for this study. The cohort comprised 215 patients classified as low to intermediate risk and 135 patients classified as high risk. Participants were categorized into two groups: the sarcopenia group (n = 143) and the non-sarcopenia group (n = 207). The study aimed to summarize the prevalence of sarcopenia among patients with localized prostate cancer and to compare the incidence of urinary incontinence immediately post-surgery, as well as at three and six months postoperatively, between the sarcopenia and non-sarcopenia groups.

**Results:**

The study assessed the prevalence of sarcopenia in a cohort of 350 patients with localized prostate cancer, revealing an overall prevalence rate of 40.86%. Specifically, the prevalence was 34.2% among patients classified as low to intermediate risk and 51.11% among those classified as high risk (P<0.01). The incidence rates of urinary incontinence were documented at various postoperative intervals: immediately after surgery, at 3 months, and at 6 months, with rates of 72%, 47.81%, and 28%, respectively. Notably, patients with sarcopenia exhibited significantly higher urinary incontinence rates compared to those without sarcopenia, at 82.52%, 65.03%, and 37.06% versus 64.73%, 35.75%, and 21.74% (P<0.01). Among low to intermediate-risk patients, the urinary incontinence rates immediately post-surgery, at 3 months, and at 6 months were 59.53%, 32.56%, and 16.28%, respectively, which were significantly lower than the rates observed in high-risk patients, recorded at 91.85%, 71.85%, and 46.67% (P<0.01). In the subgroup of low to intermediate-risk patients with sarcopenia, the postoperative urinary incontinence rates were 68.92% immediately, 54.05% at 3 months, and 18.92% at 6 months, compared to 54.61%, 21.28%, and 14.89% in patients without sarcopenia. However, the differences in incontinence rates immediately post-surgery and at 6 months were not statistically significant (P>0.05)In patients at high risk with sarcopenia, the incidence rates were 97.10%, 76.81%, and 56.52%, which were higher compared to those without sarcopenia, who exhibited rates of 86.36%, 66.67%, and 36.36%. There was no significant difference observed at three months post-surgery (P > 0.05). Regression analysis indicates that sarcopenia is significantly associated with an increased risk of urinary incontinence following radical prostatectomy at three months post-operation (OR = 0.448, 95% CI: 0.290-0.691, P < 0.001) and six months post-operation (OR = 0.175, 95% CI: 0.105-0.291, P < 0.001). After adjusting for confounding factors such as age, tumor risk stratification, diabetes, and pelvic floor function scores, sarcopenia remains an independent predictor of urinary incontinence occurrence at three months post-operation (OR = 0.320, 95% CI: 0.187-0.546, P < 0.001) and six months post-operation (OR = 0.398, 95% CI: 0.224-0.708, P = 0.002).

**Conclusions:**

Sarcopenia significantly contributes to urinary incontinence following laparoscopic radical prostatectomy and impacts the recovery process, especially in patients with high-risk prostate cancer. Evaluating muscle mass before surgery and implementing strategies to enhance it could lower the likelihood of urinary incontinence. This insight assists clinicians in improving risk evaluation and management when developing preoperative and rehabilitation strategies.

## Introduction

Prostate cancer (PCa) represents the most prevalent malignant neoplasm within the male genitourinary system and is the second most common cancer affecting men globally, surpassed only by lung cancer. According to statistics from the World Health Organization (WHO), 1.46 million new cases of prostate cancer were diagnosed in 2022, accompanied by 394,200 reported deaths (1). Radical prostatectomy (RP) is a standard therapeutic intervention for localized prostate cancer and is extensively utilized worldwide. Urinary incontinence (UI) emerges as the most frequent complication post-radical prostatectomy, with incidence rates reported between 5% and 60% (2). Research indicates that the likelihood of urinary incontinence escalates with advancing patient age, thereby substantially affecting both quality of life and confidence in treatment outcomes (3, 4).

Sarcopenia is a condition characterized by a progressive and generalized decline in skeletal muscle mass and strength, which substantially increases the risk of adverse health outcomes (5). The prevalence of sarcopenia escalates with advancing age, with statistical data indicating a prevalence of approximately 5% to 13% among individuals aged 60 to 70 years, and 11% to 50% among those aged over 80 years (6). In cancer patients, heightened protein catabolism and diminished protein synthesis significantly augment the incidence of sarcopenia (7). The prevalence of cancer-associated sarcopenia ranges from 12.5% to 72.2%, adversely impacting the quality of life of cancer patients and being closely linked to cancer prognosis (8). Meta-analyses reveal that the overall incidence of sarcopenia in patients with prostate cancer is approximately 43%, with early-stage prostate cancer patients exhibiting an incidence rate of about 31.8% (9). Research suggests that a reduction in pelvic muscle mass and strength may be associated with the onset of urinary incontinence following radical prostatectomy for prostate cancer (10). Consequently, this study was designed to investigate the prevalence of sarcopenia among patients with prostate cancer and to examine its impact on the incidence of urinary incontinence following radical prostatectomy.

## Materials and methods

### Patients

**Figure 1:**
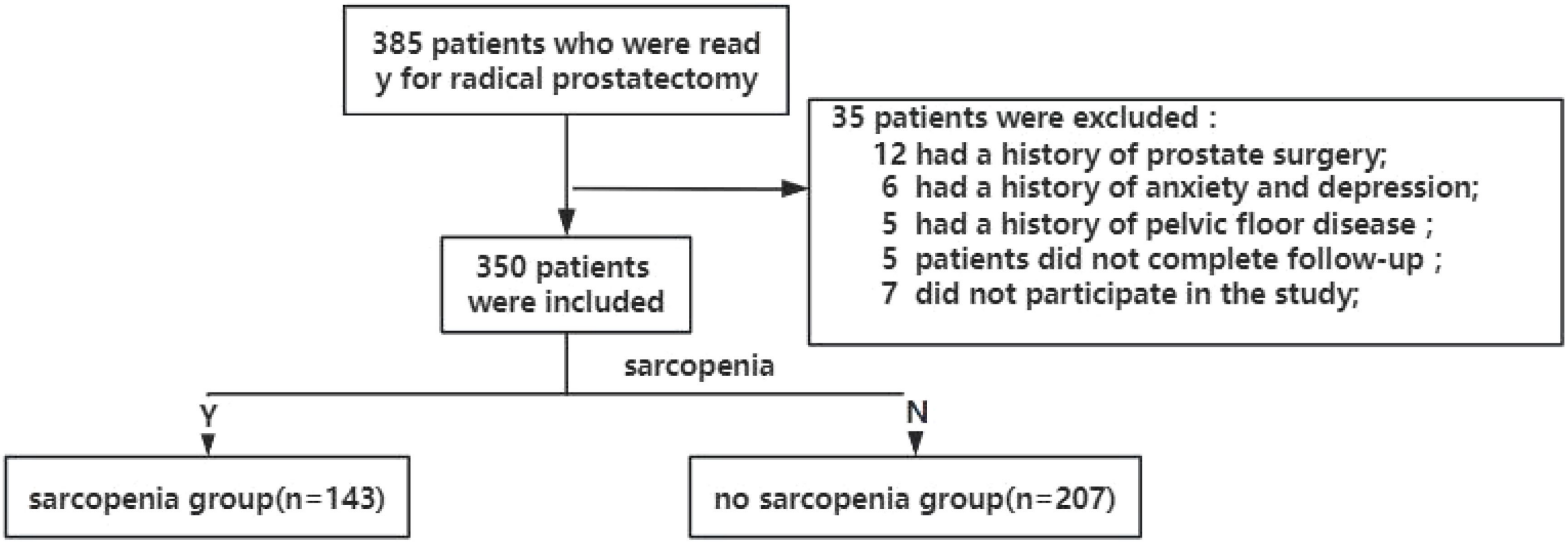
Flowchart of patients in this study.

This study was designed prospectively and received approval from the Ethics Committee of Lishui Central Hospital in Zhejiang Province, China. Initially, 385 patients diagnosed with prostate cancer were recruited, all of whom underwent laparoscopic radical prostatectomy performed by the same surgeon between January 2022 and December 2023. A postoperative follow-up period of six months was implemented, during which clinical data were systematically collected. The inclusion criteria comprised a biopsy-confirmed diagnosis of prostate adenocarcinoma, imaging evidence of localized prostate cancer, and an anticipated life expectancy exceeding ten years. Exclusion criteria encompassed a history of lower urinary tract surgery, mental illness, severe comorbidities precluding surgical tolerance, spinal cord injury or other neurological disorders, pelvic floor dysfunction, inability to comply with follow-up requirements, and unwillingness to participate in the study. Based on these criteria, 35 patients were excluded, resulting in a final cohort of 350 prostate cancer patients included in the study. The cohort of 350 patients was stratified into two groups—those with sarcopenia and those without—according to established sarcopenia criteria. Each patient underwent laparoscopic radical prostatectomy performed by the same surgeon, and postoperative outcomes, specifically the incidence of urinary incontinence and recovery status, were monitored over a six-month period. **Figure 1** illustrates the flowchart detailing the criteria for patient inclusion and exclusion in this study.

### Methods

Upon admission, all patients diagnosed with prostate cancer underwent comprehensive evaluations, including a full abdominal dual-energy computed tomography (CT) scan, a grip strength assessment, and a walking test to evaluate sarcopenia, alongside pelvic floor function tests to assess pelvic floor status. Following the exclusion of any surgical contraindications, a single surgeon conducted a non-nerve-sparing laparoscopic radical prostatectomy, incorporating routine urethral suspension and posterior urethral reconstruction. Postoperative follow-up was conducted for a duration of six months. The one-hour pad test was employed to assess the incidence of urinary incontinence immediately after the surgical procedure, as well as at three and six months postoperatively, in order to analyze the influence of sarcopenia on urinary incontinence subsequent to laparoscopic radical prostatectomy.

### Assessments

Diagnostic criteria for sarcopenia are as follows: ① Muscle mass: Skeletal muscle index at the L3 vertebra level is less than 40.8 cm²/m²; ② Muscle strength: Grip strength is less than 28 kg; ③ Physical function: Walking speed is ≤ 1 m/s.

Diagnostic categories:Presumptive sarcopenia: Criterion①; Sarcopenia: Criterion①+②or Criterion①+③; Severe sarcopenia: Criterion①+②+③ (11); Pelvic floor function assessment: The MaLiande biofeedback device(Nanjing McLand Medical Technology Co., LTD,Nanjing,China)is used to evaluate a patient’s pelvic floor function; a score of less than 80 indicates the presence of pelvic floor dysfunction, with lower scores suggesting poorer pelvic floor function.

One-hour pad test: Place a pre-weighed dry pad at the perineum; during the initial 15 minutes, the patient drinks 500ml of water and rests in bed. For the next 30 minutes, the patient walks and goes up and down stairs. In the subsequent 15 minutes, the patient should sit and stand 10 times, cough forcefully 10 times, run for 1 minute, pick up 5 small objects from the floor, and then wash hands with tap water for 1 minute. At the end of the 60-minute test, weigh the pad and instruct the patient to urinate and record the urine volume. Result interpretation: Negative: <1g, mild leakage: 2-10g, moderate leakage: 10-50g, severe leakage: >50g.

### Statistical analysis

SPSS22.0 was used to perform statistical analysis on the data. Normally distributed measurement data were expressed as mean ± standard deviation (x ± SD), independent sample t test was used for comparison between groups, and skewed distribution measurement data were expressed as median (minimum value~maximum value), using the Mann-Whitney U test. Categorical data are expressed as percentages (%) and analyzed using the x^2^ test or Fisher’s exact test. Univariable and multivariable logistic regression analyses were performed to identify influence factors of UI 3 and 6 months after surgery. P<0.05 is considered statistically significant.

## Results

### Comparison of baseline data between the two groups

There were no significant differences between the two groups in body mass index, smoking, drinking, prostate volume, education level, type of medical insurance, PSA, Gleason score, TMN staging and neoadjuvant therapy(P > 0.05).There were statistical differences in age, income, diabetes rate and pelvic floor function scores between the two groups(P<0.05).as shown in **Table 1**.

**Table 1:** Comparison of baseline data between the two groups[ ( ± SD), n (%)].

Index	Sarcopenia group (n=143)	No Sarcopenia group (n=207)	t or x^2^	P
Age (year)	75.65±7.35	74.83±7.59	2.784	0.042
BMI (kg/m2)	25.37±3.89	25.46±7.64	1.864	0.059
Education			0.054	0.718
illiteracy	13 (9.09%)	23 (11.11%)		
primary	82 (57.34%)	122 (58.94%)		
junior	33 (23.08%)	44 (21.26%)		
senior	15 (10.49%)	18 (8.69%)		
Insurance			0.322	0.56
rural	72 (50.32%)	97 (47.10%)		
urban	71 (49.68%)	110 (52.90%)		
Income (thousand)			5.001	0.025
<50	101 (70.97%)	122 (58.94%)		
50-100	32 (22.58%)	62 (29.95%)		
>100	10 (6.45%)	23 (11.11%)		
Comorbidities diabetes	35 (24.48%)	29 (14.19%)	6.200	0.013
coronary heart disease	4 (3.22%)	17 (8.39%)	3.775	0.052
cerebral infarction	8 (5.81%)	21 (10.32%)	2.132	0.144
hypertension	66 (46.45%)	89 (43.23%)	0.326	0.568
Smoking			0.298	0.585
Yes	102 (71.33%)	142 (68.59%)		
No	41 (28.67%)	65 (41.41%)		
Drinking			0.417	0.518
Yes	98 (68.53%)	135 (65.22%)		
No	45 (31.47%)	72 (34.78%)		
prostate volume (ml)	62.71±9.05	62.47±6.73	1.234	0.214
PSA (>20 ng/ml)			1.168	0.280
Yes	34 (23.78%)	60 (28.99%)		
No	109 (76.22%)	147 (71.01%)		
Gleason score (>7)			0.690	0.406
Yes	45 (31.47%)	74 (35.75%)		
No	98 (68.53%)	133 (64.25%)		
TMN staging (>T2b)			1.907	0.167
Yes	43 (30.07%)	77 (37.19%)		
No	100 (69.93%)	130 (62.81%)		
Neoadjuvant			0.08	0.778
Yes	32 (22.58%)	49 (23.87%)		
No	111 (77.42%)	158 (76.13%)		
Pelvic floor function	60.95±11.26	79.29±6.19	17.721	<0.001

BMI, Body Mass Index; PSA, prostate-specific antigen.

### The incidence of sarcopenia in prostate cancer patients with different risk stratifications

**Figure 2:**
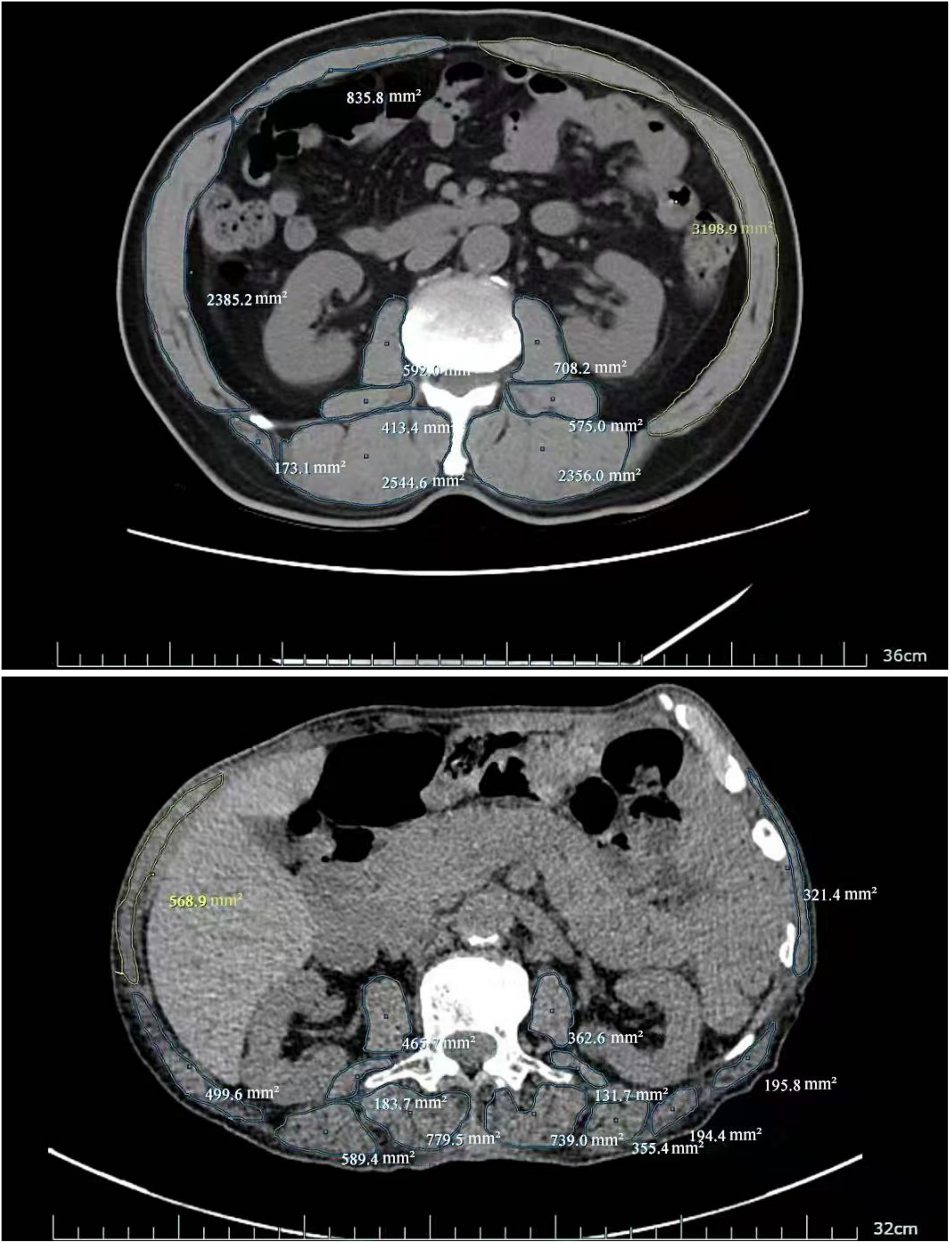
Skeletal muscle area at L3 vertebral level (No Sarcopenia vs Sarcopenia:13788.2mm^2^vs 5391.1mm^2^).

The incidence of sarcopenia among all prostate cancer patients is 40.86%. Among them, the incidence of sarcopenia in patients with low to intermediate-risk prostate cancer is 34.42%, while it is 51.11% in high-risk prostate cancer patients. There is a statistically significant difference in the incidence of sarcopenia among prostate cancer patients with different risk stratifications (P<0.05). See **Table 2** and **Figure 2** for details.

**Table 2:** Incidence of sarcopenia in prostate cancer with different risk stratifications [ (n (%)].

Index	Prostate cancer	Low and medium risk prostate cancer (n=215)	High risk prostate cancer (n=135)	x2	P
Sarcopenia	143 (40.86%)	74 (34.42%)	69 (51.11%)	9.563	0.002
Non-sarcopenia	207 (59.14%)	141 (65.58%)	66 (48.89%)		

### Comparison of occurrence of urinary incontinence between two groups

**Figure 3:**
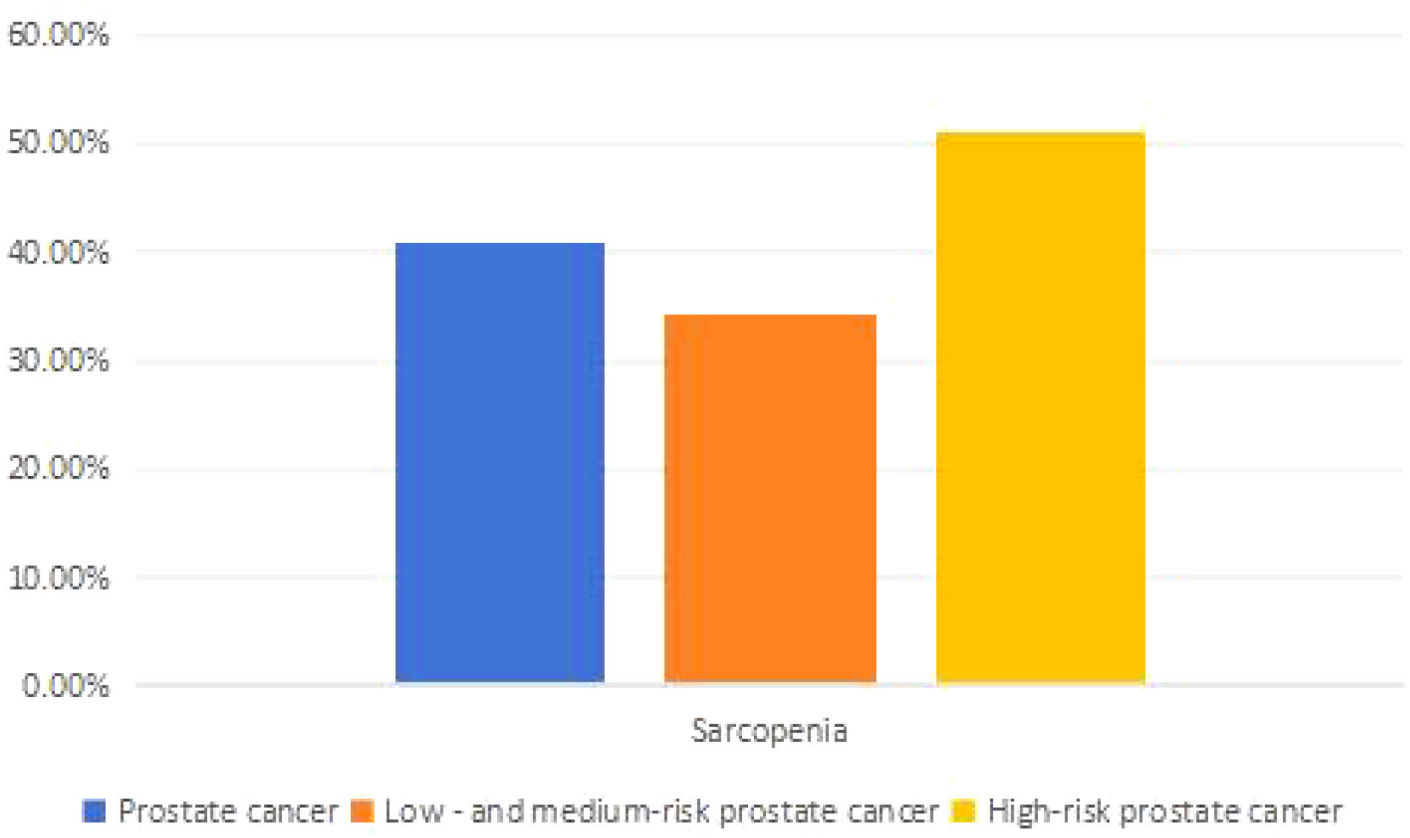
Occurrence of urinary incontinence in different risk prostate cancer.

The postoperative urinary incontinence rates for all patients immediately, at 3 months, and at 6 months were 72%, 47.81%, and 28%, respectively. Among sarcopenia patients, the rates were 82.52%, 65.02%, and 37.06%, while for non-sarcopenia patients, the rates were 64.73%, 35.75%, and 21.74%, respectively. The incidence of postoperative urinary incontinence in sarcopenia patients was significantly higher than in non-sarcopenia patients (P<0.05). For more details, see **Table 3** and **Figure 3**.

**Table 3:** Comparison of postoperative urinary incontinence between the two groups (n (%)].

Time	All patients	Sarcopenia group (n=143)	No Sarcopenia group (n=207)	x2	P
immediately	252 (72%)	118 (82.52%)	134 (64.73%)	13.267	<0.001
3 months	167 (47.81%)	93 (65.03%)	134 (64.73%)	29.076	<0.001
6 months	98 (28%)	53 (37.06%)	45 (21.74%)	9.851	0.002

### Comparison of postoperative urinary incontinence in patients with different risk stratified prostate cancer

**Figure 4:**
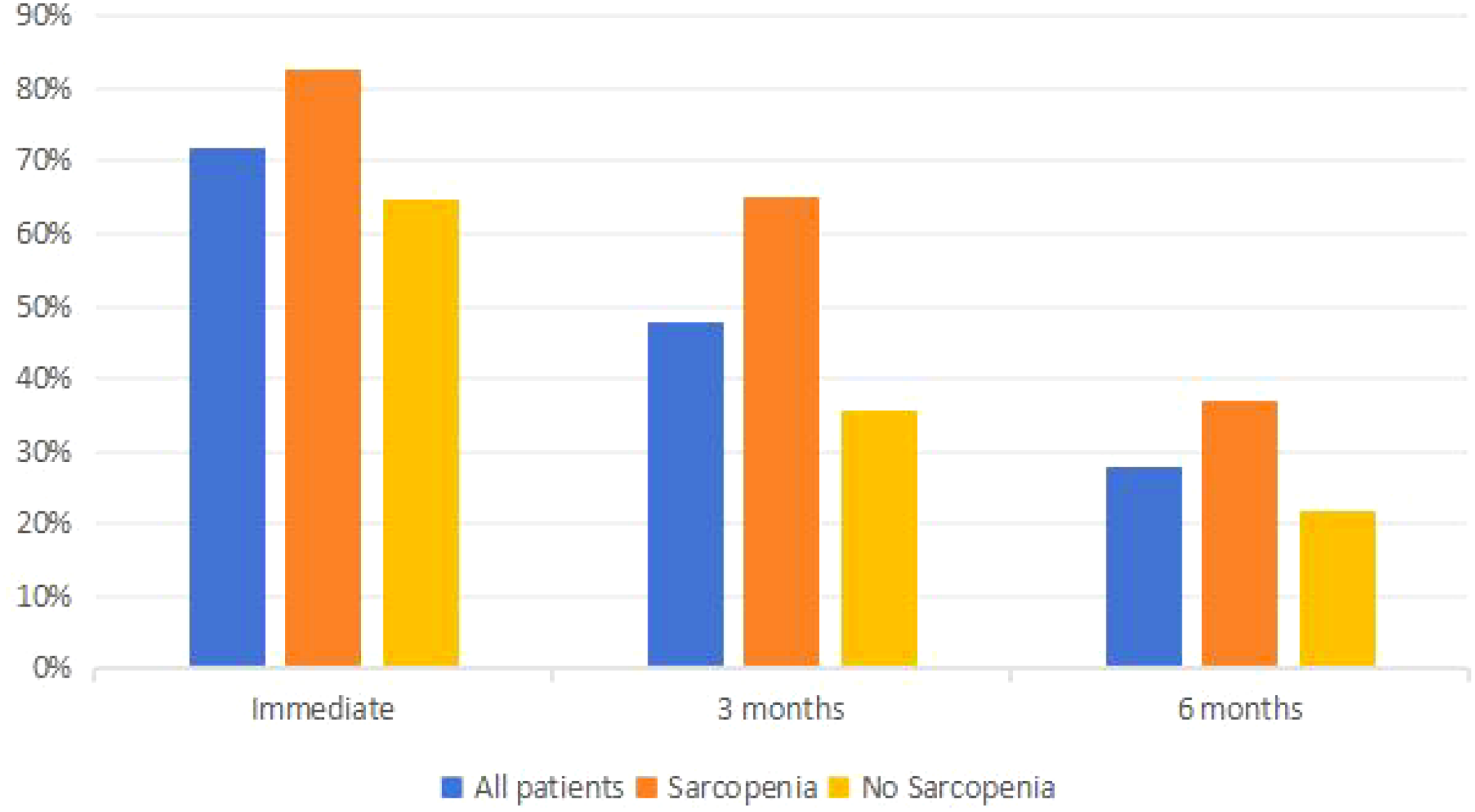
Occurrence of urinary incontinence in both groups.

**Figure 5:**
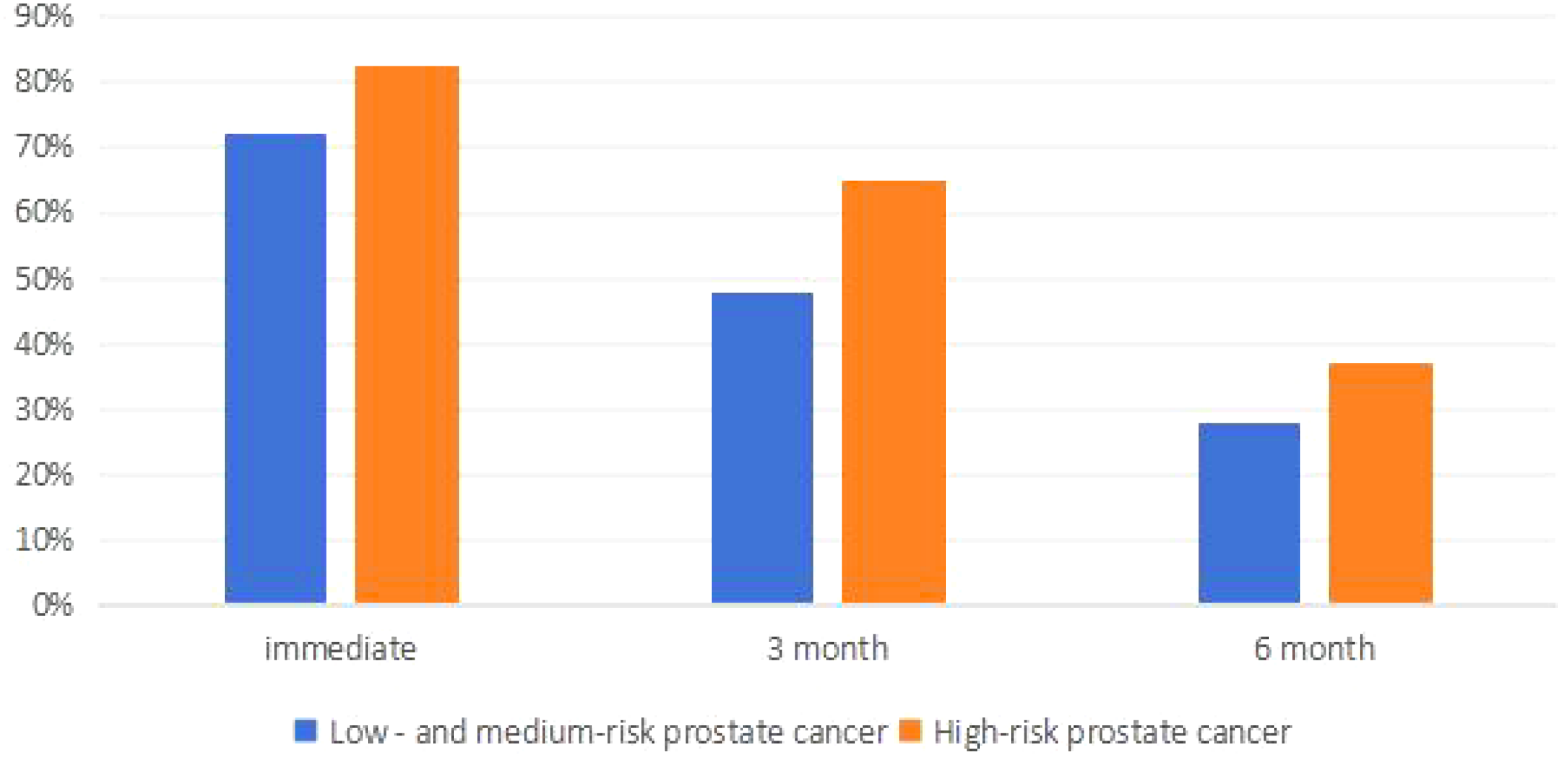
Comparison of postoperative urinary incontinence in patients with different risk stratified prostate cancer.

The incidence of urinary incontinence immediately post-operatively and at 3 months and 6 months for patients with low to intermediate-risk prostate cancer was 72%, 47.81%, and 28%, respectively, whereas for high-risk prostate cancer patients, it was 82.52%, 65.03%, and 37.06%, respectively, significantly higher than that of low to intermediate-risk patients (P<0.05). Refer to **Table 4** and **Figure 4** for details. Among low to intermediate-risk prostate cancer patients with sarcopenia, the incidence of urinary incontinence immediately post-operatively and at 3 months was significantly higher at 68.92% and 54.05% compared to those without sarcopenia, which was 54.61% and 21.28% (P<0.05). However, there was no significant difference in the incidence of urinary incontinence between the two groups at 6 months post-operatively, being 18.92% and 14.89%, respectively (P>0.05). For high-risk patients with sarcopenia, the incidence of urinary incontinence immediately post-operatively and at 6 months was higher compared to those without sarcopenia, at 97.10%, 56.52% and 86.36%, 36.36%, respectively (P<0.05) (**Figure 5** and **Table 5**).

**Table 4:** Comparison of postoperative urinary incontinence in patients with different risk stratified prostate cancer [(n(%)].

Time	Low and medium risk prostate cancer (n=215)	High risk prostate cancer (n=135)	x2	P
immediately	128 (59.53%)	124 (91.85%)	42.961	<0.001
3 months	70 (32.56%)	97 (71.85%)	51.324	<0.001
6 months	35 (16.28%)	63 (46.67%)	37.984	<0.001

**Table 5:** Comparison of postoperative urinary incontinence in patients with different risk stratified prostate cancer complicated with sarcopenia [ (n (%)].

Time	Low and medium risk with sarcopenia (n=74)	Low and medium risk without sarcopenia (n=141)	x2	P	High risk with sarcopenia (n=69)	High risk without sarcopenia (n=66)	x2	P
immediately	51 (68.92%)	77 (54.61%)	4.125	0.049	67 (97.10%)	57 (86.36%)	5.197	0.023
3 months	40 (54.05%)	30 (21.28%)	23.745	0.001	53 (76.81%)	44 (66.67%)	1.717	0.190
6 months	14 (18.92%)	21 (14.89%)	0.577	0.448	39 (56.52%)	24 (36.36%)	5.507	0.019

### Analysis of risk factors of urinary incontinence after radical prostate cancer surgery

Univariate and multivariate logistic regression analyses for postoperative urinary incontinence at 3 and 6 months showed that at 3 months, high-risk prostate cancer, sarcopenia, and pelvic floor function scores were risk factors for urinary incontinence after radical prostatectomy. At 6 months, univariate analysis revealed high-risk prostate cancer, sarcopenia, pelvic floor function scores, diabetes, and advanced age as risk factors, while multivariate analysis identified sarcopenia, pelvic floor function scores, and diabetes as independent risk factors for urinary incontinence. See **Tables 6**, **7** for details.

**Table 6:** Logistic regression analysis of risk factors for urinary incontinence at 3 months after surgery.

Influencing factor	Univariable analysis			Multivariable analysis		
	OR	95%CI	P	OR	95%CI	P
High-risk prostate cancer	0.358	0.231-0.556	<0.001	0.384	0.244-0.604	<0.001
sarcopenia	0.448	0.290-0.691	<0.001	0.320	0.187-0.546	<0.001
Age > 70 years	1.084	0.712-1.650	0.708			
Smoking	0.890	0.585-1.354	0.586			
Drinking	0.817	0.537-1.243	0.345			
BMI>25kg/m^2^	0.742	0.484-1.138	0.172			
Prostate volume > 60ml	0.888	0.582-1.354	0.581			
hypertension	0.890	0.585-1.354	0.586			
diabetes	0.946	0.622-1.440	0.796			
Hyperlipidemia	1.084	0.712-1.650	0.708			
Pelvic floor function score	0.852	0.823-0.883	<0.001	0.970	0.950-0.991	0.005

BMI, Body Mass Index.

**Table 7:** Logistic regression analysis of risk factors for urinary incontinence at 6 months after surgery.

Influencing factor	Univariable analysis			Multivariable analysis		
	OR	95%CI	P	OR	95%CI	P
High-risk prostate cancer	0.539	0.336-0.864	0.01	0.635	0.370-1.088	0.098
sarcopenia	0.175	0.105-0.291	<0.001	0.398	0.224-0.708	0.002
Age > 70 years	0.499	0.307-0.809	0.005	0.525	0.300-0.919	0.064
Smoking	0.650	0.406-1.040	0.073			
Drinking	0.715	0.447-1.144	0.162			
BMI>25kg/m^2^	0.872	0.541-1.406	0.575			
Prostate volume > 60ml	1.142	0.715-1.824	0.679			
hypertension	0.650	0.406-1.040	0.073			
diabetes	0.427	0.262-0.696	0.001	0.523	0.301-0.910	0.022
Hyperlipidemia	0.499	0.307-0.809	0.075			
Pelvic floor function score	1.099	1.067-1.132	<0.001	0.855	0.825-0.885	<0.001

BMI, Body Mass Index.

## Discussion

Laparoscopic radical prostatectomy is a primary therapeutic intervention for localized prostate cancer. A significant postoperative complication of this procedure is urinary incontinence, which adversely impacts patients’ quality of life and can induce anxiety and concern among patients and their families, potentially leading to doubts regarding the surgery’s overall efficacy (12). The etiology of postoperative urinary incontinence is multifaceted, with numerous studies indicating that factors such as tumor staging, surgical techniques, preservation of pelvic floor structures, and retention of functional urethra influence its incidence (13–16). Sarcopenia, a condition prevalent among cancer patients, significantly impairs skeletal muscle function and has been shown in multiple studies to affect the entire continuum of prostate cancer treatment, particularly in advanced stages (17, 18). Does sarcopenia affect urinary incontinence following radical prostate cancer surgery? Our study examined the presence of sarcopenia in prostate cancer patients and its impact on urinary incontinence post-surgery. Our study identified that 40.86% of prostate cancer patients exhibited sarcopenia, a finding consistent with previous research, where meta-analyses have reported an overall sarcopenia prevalence of approximately 43% among prostate cancer patients, and an incidence rate of around 31.8% in early-stage cases (9). Comparative analysis between prostate cancer patients with and without sarcopenia revealed no statistically significant differences in tumor characteristics, body mass index (BMI), educational attainment, type of medical insurance, smoking and alcohol consumption history, or the presence of coronary heart disease and hyperlipidemia. Nonetheless, individuals in the sarcopenia cohort were generally older, had lower income levels, exhibited a higher prevalence of diabetes, and demonstrated reduced pelvic floor function scores compared to those without sarcopenia. Literature (19) suggests that the incidence of sarcopenia escalates with advancing age, and socioeconomic status may influence nutritional intake, thereby affecting sarcopenia prevalence. Additionally, diabetes has been implicated in the deterioration of muscle function, thereby exacerbating sarcopenia. Our findings corroborate existing evidence (20) suggesting that sarcopenia adversely impacts pelvic floor muscle function.

In this study, the overall incidence of sarcopenia among prostate cancer patients was found to be 40.86%. Notably, the incidence was significantly elevated in patients with high-risk prostate cancer compared to those with medium or low risk, aligning with findings from previous research. Several studies (8, 9) have indicated that the prevalence of sarcopenia is markedly higher in patients with advanced-stage tumors compared to those with early-stage tumors. This phenomenon may be attributed to the progressive catabolic effects of the tumor, which result in a reduction of the patient’s muscle mass. Longitudinal studies on prostate cancer patients have demonstrated that, as the disease advances, the probability of developing sarcopenia increases substantially during the course of treatment.

In this study, we observed that the incidence of immediate urinary incontinence following radical prostatectomy was as high as 72% among all patients. However, with the progression of postoperative time, symptoms of urinary incontinence showed gradual improvement, with the incidence decreasing to approximately 28% at six months post-surgery, aligning with findings from previous studies (12). Some researchers have employed robot-assisted radical prostatectomy, achieving long-term postoperative urinary incontinence rates of less than 10% (21). We conducted a further analysis of patients based on the presence of sarcopenia. Patients with prostate cancer who also had sarcopenia exhibited higher rates of both immediate postoperative urinary incontinence and incontinence at six months compared to those without sarcopenia. This suggests that sarcopenia increases the likelihood of urinary incontinence following radical prostatectomy, corroborating previous research findings (22). In patients with low to intermediate-risk prostate cancer, the presence of sarcopenia is associated with a significantly increased incidence of short-term postoperative urinary incontinence compared to those without sarcopenia. However, at six months postoperatively, the prevalence of urinary incontinence converges between the two groups, suggesting that sarcopenia does not have a significant long-term impact on urinary incontinence in this patient cohort, although it does adversely affect short-term urinary control recovery following surgery. Conversely, in patients with high-risk prostate cancer, those with sarcopenia exhibit a consistently higher incidence of urinary incontinence at all evaluated time points compared to their non-sarcopenic counterparts. This observation implies that sarcopenia exerts a more pronounced influence on urinary incontinence in high-risk patients, potentially due to the more extensive surgical resection required in this group, which may compromise pelvic floor muscle function, thereby increasing the likelihood of postoperative urinary incontinence and impeding the restoration of urinary function.

In this study, we conducted an analysis of the risk factors associated with postoperative urinary incontinence among all patients. At three months following surgery, both univariate and multivariate regression analyses identified high-risk prostate cancer, sarcopenia, and pelvic floor dysfunction as independent risk factors for urinary incontinence. In comparison to medium- and low-risk prostate cancer, high-risk prostate cancer necessitates a more extensive surgical excision, which results in greater disruption to the pelvic floor structure and function, thereby elevating the risk of postoperative urinary incontinence. At six months following surgery, our analysis of risk factors for urinary incontinence identified, through univariate analysis, that high-risk prostate cancer, sarcopenia, age over 70, diabetes, and pelvic floor dysfunction were associated with increased risk. However, multivariate analysis revealed that only sarcopenia, diabetes, and pelvic floor dysfunction emerged as independent risk factors, whereas prostate cancer risk stratification and age did not significantly influence the long-term incidence of urinary incontinence. Diabetes is known to affect peripheral vascular and nerve function, which in turn can impair skeletal muscle function. Research indicates that skeletal muscle function deteriorates more rapidly in diabetic patients compared to non-diabetic individuals (23). Sarcopenia has a substantial impact on the function and recovery of pelvic floor muscles. The pelvic floor function score serves as a quantitative measure of pelvic muscle function, with the score reflecting the condition of pelvic muscle function and influencing the likelihood of postoperative urinary incontinence.

The study investigated the incidence of urinary incontinence following laparoscopic radical prostatectomy and examined the associated risk factors. The findings identified sarcopenia as an independent risk factor for persistent postoperative urinary incontinence. Nonetheless, the analysis process presented certain limitations. Specifically, the severity of sarcopenia was not stratified, leaving it unclear whether varying degrees of sarcopenia exert differential impacts on the incidence of urinary incontinence. Furthermore, the factor analysis concerning the risk of urinary incontinence lacked comprehensiveness, potentially influencing the study’s outcomes. Despite these limitations, the data collection and analysis were executed with rigor, rendering the findings reliable. The recognition of sarcopenia as an independent risk factor for urinary incontinence post-radical prostatectomy can inform strategies for the prevention and management of urinary incontinence in this patient population.

In conclusion, this study determined that approximately 40% of prostate cancer patients exhibit sarcopenia, with a notably higher prevalence among those with high-risk prostate cancer compared to individuals with low to medium risk. Sarcopenia serves as an independent risk factor impacting the incidence of urinary incontinence following radical prostatectomy and influences the recovery trajectory of incontinence. Consequently, In patients with sarcopenia, implementing perioperative rehabilitation strategies may reduce the incidence of postoperative incontinence, bearing significant clinical implications for improving treatment efficacy and the quality of life for patients experiencing urinary incontinence after laparoscopic radical prostatectomy.

## Data Availability

The original contributions presented in the study are included in the article/supplementary material. Further inquiries can be directed to the corresponding author.
